# Nottingham Children's Hospital

**Published:** 1899-02-18

**Authors:** Lettice A. Floyd

**Affiliations:** Sister of the Nottingham Children's Hospital from September, 1895, to September, 1898


### NOTTINGHAM CHILDREN'S HOSPITAL.

Miss Lettice A. Floyd, Sister of the Nottingham Children's Hospital from September, 1895, to September, 1898, writes: Seeing Miss Catherine Ada Morrish's repeated statement in your paper of February 11th that she had never been taught to give enemata before taking a case at isolation, I feel it only just to write and say that Miss Catherine Ada Morrish was my probationer when she first came to the Nottingham Children's Hospital, and I taught her myself to give several enemata in my ward, both letting her see me give one and also seeing her give them herself. I am only refuting one "inaccuracy" out of many in Miss Catherine Ada Morrish's statement, but I should like to add that during five years' work at the Nottingham Children's Hospital I frequently worked at isolation, and found it quite possible to live a comfortable, clean, and refined life there, and that neither the nurses nor the patients were ever neglected in any particular. Trusting that you will find a corner for this letter in your next issue.

